# Cranial POCUS in Neonates and Infants: Structured Evidence Synthesis and Proposal of the KORE Brain POCUS Protocol

**DOI:** 10.24908/pocusj.v11i01.20031

**Published:** 2026-04-22

**Authors:** Salvatore Michele Carnazzo, Desirèe Balconara, Salvatore Scirè Calabrisotto, Andrea Domenico Praticò

**Affiliations:** 1 PhD Program in Innovative Technologies in Biomedical Sciences, Faculty of Medicine and Surgery, Kore University of Enna, Enna, Italy; 2 Pediatric Pneumoallergology Unit, Department of Pediatrics, A.O.U. Policlinico San Marco, University of Catania, Catania, Italy; 3 Department of Clinical and Experimental Medicine, University of Catania, Catania, Italy; 4 Department of Pediatrics, Faculty of Medicine and Surgery, Kore University of Enna, Enna, Italy

**Keywords:** Point-of-Care Ultrasound, POCUS, Cranial Ultrasonography, Pediatrics, Neonates, Neurosonography, Transcranial Doppler, Intracranial Hemorrhage, Critical Care

## Abstract

**Background:**

Cranial point of care ultrasound (POCUS) is an emerging bedside tool for rapid evaluation of neurological emergencies in neonates and infants. Although widely used in neonatal intensive care, its application in emergency and critical care remains heterogeneous and lacks standardized protocols.

**Objective:**

To synthesize the current literature on cranial POCUS in neonates and infants and propose a structured clinical protocol (KORE Brain POCUS Protocol) to support early diagnosis and management in emergency and critical care settings.

**Methods:**

A structured search of PubMed, Scopus, Web of Science, and Cochrane Library (through August 2025) identified studies on cranial POCUS in neonatal and infant acute care. Eligible studies included original research, observational studies, case series, and narrative or systematic reviews. Two reviewers independently screened and extracted data on study characteristics, clinical context, and feasibility.

**Results:**

Nine studies met the inclusion criteria. Cranial POCUS was consistently reported as feasible for detecting intracranial hemorrhage (ICH), ventricular dilatation, sinovenous thrombosis, and posterior fossa lesions, and for assessing cerebral hemodynamics using transcranial Doppler. Based on converging findings, we developed the KORE Brain POCUS Protocol, a stepwise framework integrating standardized ultrasound windows and Doppler assessment for pediatric emergency and critical care.

**Conclusions:**

Cranial POCUS is a promising, rapid, noninvasive tool for the bedside evaluation of acute neurological conditions in neonates and infants. The proposed KORE Brain POCUS Protocol provides a structured approach to support early diagnosis and clinical decision-making. Prospective studies are needed to validate its diagnostic performance and clinical impact.

## Introduction

Point of care ultrasound (POCUS) refers to diagnostically oriented ultrasonography performed and interpreted at the bedside by the treating clinician, enabling immediate, data-informed decisions without reliance on remote radiology support [1]. In emergency and critical care settings, POCUS has revolutionized patient evaluation with established protocols such as Focused Assessment with Sonography for Trauma (FAST) and Extended Focused Assessment with Sonography for Trauma (eFAST) for trauma, Rapid Ultrasound in Shock and Hypotension (RUSH) for circulatory failure, and Bedside Lung Ultrasound in Emergency (BLUE) for undifferentiated respiratory distress [2–4]. However, these protocols are primarily developed for adults and do not always translate to pediatric or neonatal patients, where anatomical and physiological differences—such as smaller size, open fontanelles, and distinct pathologies—necessitate tailored approaches [5]. POCUS is already routinely used in neonatal intensive care settings, but its structured application in pediatric emergency care remains limited [6]. In neonatal and pediatric care, cranial ultrasound offers unique advantages due to the presence of patent fontanelles, which serve as excellent acoustic windows [7]. This enables non-invasive, rapid visualization of intracranial structures, including ventricles, hemorrhages, hydrocephalus, midline shifts, and even cerebral blood flow when combined with Doppler modalities without sedation or radiation exposure [4]. Despite these benefits, there is no standardized “brain POCUS” protocol currently integrated into pediatric emergency pathways, unlike well-established adult POCUS protocols. This gap is noteworthy given the increasingly recognized utility of cranial POCUS in detecting critical pathologies in neonates, such as acute intraventricular hemorrhage (IVH), hydrocephalus progression, or midline shift, with implications for neurodevelopmental outcomes and timely interventions [5]. The intended clinical application of brain POCUS in emergency and critical care is focused on time-critical presentations in neonates and young infants, including seizures, bulging fontanelle with suspected hydrocephalus, selected head trauma, and suspicion of IVH or ventricular enlargement. The protocol is designed as an early bedside triage framework rather than a replacement for definitive neuroimaging. This article aims to (1) review the rationale and current state of cranial POCUS in pediatric critical care, (2) highlight the absence of emergency-focused protocols for brain ultrasound in neonates and children, and (3) propose a structured and scalable brain POCUS protocol tailored for pediatric emergency and critical care, tentatively titled the “KORE Brain POCUS Protocol.”

## Methods

### Study Design

**Figure 1.**
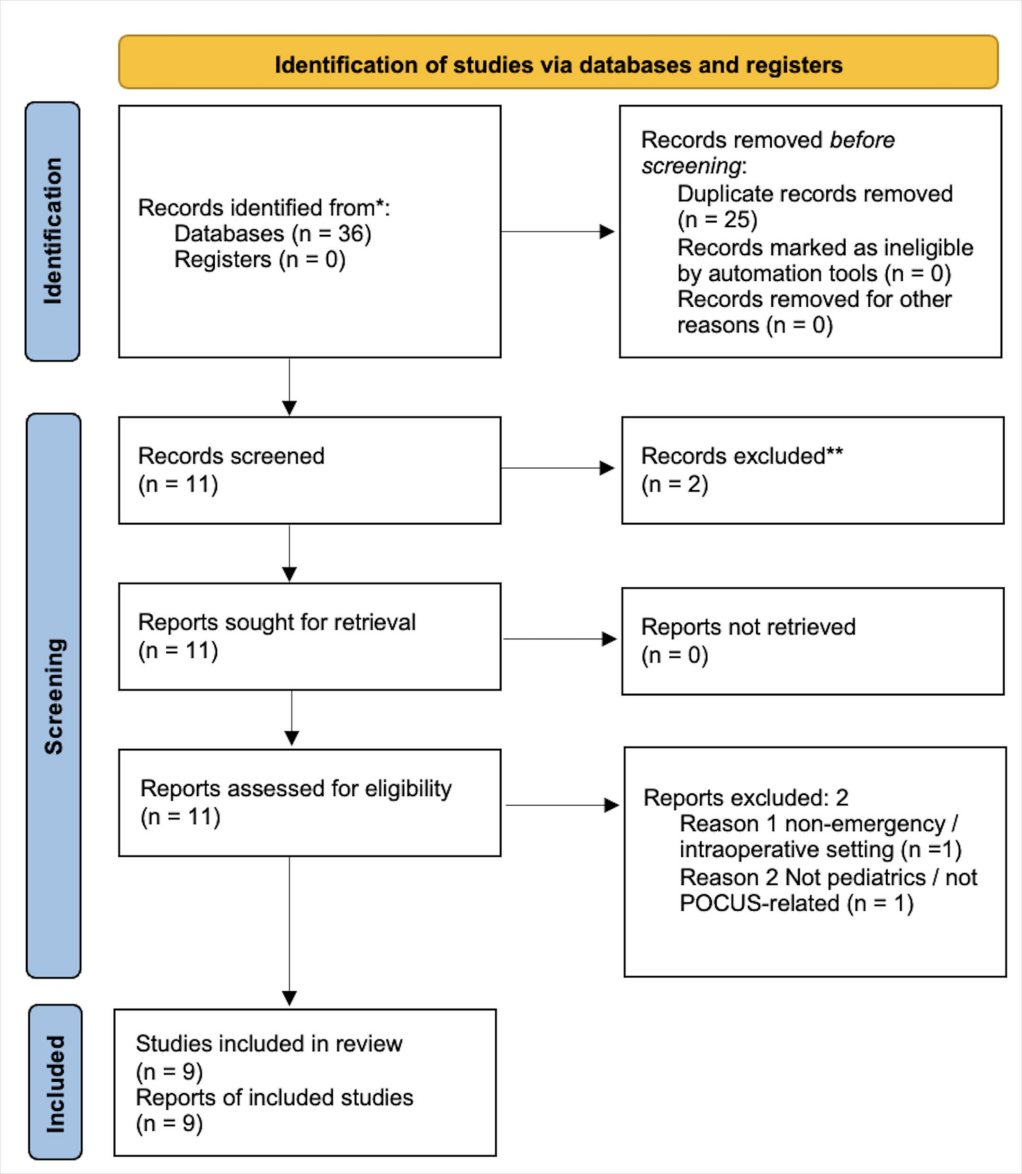
PRISMA 2020 flow diagram of study selection summarizing the identification, screening, eligibility assessment, and inclusion of studies for this structured evidence synthesis. Adapted from Page MJ et al. BMJ. 2021;372:n71, licensed under CC BY 4.0 [8].

This review is an evidence-informed, structured literature review that follows the core principles of the PRISMA 2020 statement (Figure 1) [8]. The primary objective was to synthesize current evidence on the use of cranial POCUS in neonatal and pediatric emergency and critical care settings, with particular emphasis on diagnostic and monitoring applications. Given the limited and heterogeneous nature of the available studies, data were synthesized narratively rather than pooled quantitatively.

### Search Strategy

A comprehensive search included literature from PubMed/MEDLINE, Embase, Scopus, and Web of Science from inception until August 20, 2025. The following Boolean search strings were applied, tailored to each database syntax:

PubMed:((“point-of-care ultrasound”[tiab] OR POCUS[tiab] OR “bedside ultrasound”[tiab] OR “bedside imaging”[tiab] OR “point-of-care imaging”[tiab]) AND (“cranial ultrasound”[tiab] OR neurosonography[tiab] OR “transfontanelle ultrasound”[tiab] OR “brain ultrasound”[tiab]) AND (neonate*[tiab] OR newborn*[tiab] OR infant[tiab] OR pediatric[tiab] OR child[tiab]) AND (“emergency”[tiab] OR “acute”[tiab] OR “critical”[tiab] OR “resuscitation”[tiab] OR “intensive care”[tiab]))

Scopus (TITLE-ABS-KEY):TITLE-ABS-KEY(“point-of-care ultrasound” OR POCUS OR “bedside ultrasound” OR “bedside imaging” OR “point-of-care imaging”)AND TITLE-ABS-KEY(“cranial ultrasound” OR neurosonography OR “transfontanelle ultrasound” OR “brain ultrasound”)AND TITLE-ABS-KEY(neonate* OR newborn* OR infant OR pediatric OR child*) AND TITLE-ABS-KEY(emergency OR acute OR critical OR resuscitation OR “intensive care”)

Web of Science (TS field):TS=(“point-of-care ultrasound” OR POCUS OR “bedside ultrasound” OR “bedside imaging” OR “point-of-care imaging”) AND TS=(“cranial ultrasound” OR neurosonography OR “transfontanelle ultrasound” OR “brain ultrasound”) AND TS=(neonate* OR newborn* OR infant OR pediatric OR child) AND TS=(“emergency” OR “acute” OR “critical” OR “resuscitation” OR “intensive care”)

Embase (Title/Abstract): ((TI “point-of-care ultrasound” OR AB “point-of-care ultrasound” OR TI POCUS OR AB POCUS OR TI “bedside ultrasound” OR AB “bedside ultrasound” OR TI “bedside imaging” OR AB “bedside imaging” OR TI “point-of-care imaging” OR AB “point-of-care imaging”))AND((TI “cranial ultrasound” OR AB “cranial ultrasound” OR TI neurosonography OR AB neurosonography OR TI “transfontanelle ultrasound” OR AB “transfontanelle ultrasound” OR TI “brain ultrasound” OR AB “brain ultrasound”)) AND ((TI neonate* OR AB neonate* OR TI newborn* OR AB newborn* OR TI infant OR AB infant OR TI pediatric OR AB pediatric OR TI child OR AB child)) AND ((TI emergency OR AB emergency OR TI acute OR AB acute OR TI critical OR AB critical OR TI resuscitation OR AB resuscitation OR TI “intensive care” OR AB “intensive care”))

The search was supplemented by hand-screening of reference lists from included articles and relevant reviews.

Studies were eligible for inclusion if they met the following criteria:

Population: Neonates and children (≤18 years).

Intervention: Cranial ultrasound performed as POCUS (transfontanelle, transtemporal, or other bedside approaches).

Setting: Emergency department, neonatal intensive care unit (NICU) or pediatric intensive care unit (PICU), or perioperative critical care.

Outcomes: Diagnostic accuracy or clinical utility in detecting conditions such as IVH, hydrocephalus, elevated intracranial pressure (ICP), or ischemia, as well as feasibility in acute care scenarios.

Study types: Randomized trials, prospective or retrospective cohorts, cross-sectional studies, case series (≥5 patients), and systematic or narrative reviews relevant to brain POCUS.

Exclusion criteria included studies limited to adult populations, preclinical or technical-only research without clinical application, non-peer-reviewed reports, non-English language publications, and abstract-only sources.

### Study Selection

Two reviewers independently screened titles and abstracts using Rayyan QCRI. Full texts of potentially eligible studies were retrieved and assessed against the inclusion criteria. Discrepancies were resolved by discussion or consultation with a third reviewer.

### Data Extraction

A standardized data extraction sheet was applied in Excel, recording: first author, year, title, journal; study type, setting, population, sample size; comparator (computed tomography (CT), magnetic resonance imaging (MRI), standard ultrasound); main outcome(s); key findings and relevance to cranial POCUS Extraction was performed independently by two reviewers.

### Risk of Bias Assessment

The methodological quality of the two observational studies was qualitatively appraised using the Newcastle-Ottawa Scale framework [9]. Evaluation focused on three domains: patient selection, comparability, and outcome assessment. Case reports and the narrative review were not formally assessed with standardized tools; however, their inherent methodological limitations, such as small sample size, lack of control groups, and high risk of selection and reporting bias, were acknowledged.

### Data Synthesis

Due to heterogeneity in populations, interventions, and outcomes, results were synthesized narratively and tabulated. Key domains of brain POCUS application were identified and mapped. This synthesis informed the development of a conceptual clinical protocol (KORE Brain POCUS Protocol).

## Results

### Study Selection and Characteristics

The search across databases yielded 36 records. After removing 25 duplicates, 11 records were screened in full; 2 were excluded at eligibility (non-emergency intraoperative focus; not pediatric/POCUS), leaving 9 studies for inclusion. Designs were heterogeneous: 3 narrative reviews/overview pieces [10-12], 1 retrospective case series [13], 1 prospective pilot study in the emergency department [14], 1 cross-sectional cohort in the NICU [15], and 3 single-patient case reports [16–18]. Settings spanned the NICU/PICU (most studies), with one pediatric emergency department cohort [14]. Across the primary studies, a total of 64 neonates/infants were directly evaluated with brain POCUS (n = 6 in the emergency department pilot; n=5 in the neonatal procedure series; n=52 in the NICU cohort; plus 3 single cases).

### Populations, Scanning Windows, and Operators

All included works focused on neonates and young infants with open fontanelles, except Rowland (2020), which also covered older children for transcranial Doppler [11]. The anterior fontanelle was the primary window in every bedside series; mastoid/posterolateral and posterior fossa windows were emphasized for posterior fossa and midline lesions [17,18]. Reviews highlighted the role of transcranial Doppler via the anterior fontanelle and transtemporal windows to interrogate waveforms from the anterior cerebral artery (ACA)/middle cerebral artery (MCA)/internal carotid artery (ICA)/basilar arteries (BA) [10,11]. Examinations were performed by neonatologists, pediatric emergency physicians, or intensivists after targeted training [14,15].

### Risk of Bias

According to the Newcastle-Ottawa framework [9], both McCormick (2015) [14] and Cizmeci (2018) [13] demonstrated adequate patient selection and outcome assessment through standardized neuroimaging and clinical follow-up. Neither study controlled for potential confounders, resulting in an overall moderate risk of bias. Case reports and the narrative review were considered to have a high inherent risk of bias due to their design limitations.

### Diagnostic Applications and Performance

#### Intracranial Hemorrhage (ICH) and Acute Intracranial Pathology

In the only prospective pilot study conducted in the emergency department, McCormick (2015) evaluated six infants aged ≤3 months presenting with acute neurological concerns [14]. Bedside cranial ultrasound performed by pediatric emergency physicians successfully identified ICH in real time, with complete concordance to radiology findings. This approach avoided the need for sedation or patient transport and contributed to a reduction in CT utilization. Additional diagnostic evidence was provided by several case reports. Halm (2011) described an extremely low birth weight neonate with *E. coli* meningitis in whom brain POCUS detected evolving parenchymal injury, enabling early intervention [16]. Similarly, Oulego-Erroz (2020) [17] and Lange (2021) [18] reported cases of diffuse intrinsic midline glioma in which the use of mastoid and posterolateral windows was decisive for early suspicion and referral, highlighting the incremental diagnostic value of extended ultrasound windows [19,20]. At a systems level, two narrative reviews emphasized the synergistic role of grayscale cranial ultrasound combined with transcranial Doppler in the early detection of hydrocephalus, midline shift, and hemodynamic compromise [10,11]. These sources underline the potential of brain POCUS to support rapid, bedside neurological assessment in resource-limited or time-sensitive scenarios.

#### Post-Hemorrhagic Ventricular Dilatation (PHVD)/Hydrocephalus

A recent overview by Dan (2025) consolidated the normative and threshold metrics most commonly applied for the surveillance of PHVD in preterm neonates [12]. In particular, the ventricular index (VI), anterior horn width (AHW), and ratios such as the frontal–occipital horn ratio (FOHR), defined as the ratio between the combined widths of the frontal and occipital horns and the biparietal diameter, and the frontal–temporal horn ratio (FTHR), defined as the ratio between the frontal and temporal horn widths, were highlighted as reliable, reproducible parameters. The review emphasized that these measurements, when obtained serially and plotted over time, can serve as early indicators for neurosurgical referral, thereby directly informing brain POCUS protocols in this population. Additional evidence comes from the cross-sectional cohort study by Yengkhom (2021), which evaluated 52 neonates undergoing late-onset sepsis assessments [15]. In this cohort, cranial ultrasound abnormalities, including ventriculomegaly, IVH, and periventricular leukomalacia (PVL), were identified in 4 of 52 patients (7.7%). These findings contributed meaningfully to clinical decision-making, complementing lung and abdominal POCUS within an integrated multi-organ evaluation pathway.

#### Hemodynamic Monitoring (Transcranial Doppler)

Reviews detail fontanelle transcranial Doppler as feasible for real-time assessment of cerebral perfusion (peak systolic/end-diastolic velocity; pulsatility index (PI) / resistive index (RI)), autoregulation, and suspected raised ICP in critically ill children [10,11]. These syntheses provide age-aware interpretations and practical scanning tips relevant to emergency pathways.

#### Procedural Guidance and Impact on Management

The study by Cizmeci (2018) provided one of the few structured evaluations of procedural POCUS in the neonatal neurocritical setting [13]. In this small case series involving five neonates (three with intra-axial and two with extra-axial hemorrhages), ultrasound guidance enabled safe percutaneous needle aspiration directly at the bedside. Procedural success was achieved in all cases, with no infectious complications reported. Importantly, cranial ultrasound was used not only to guide patient selection but also to monitor the immediate post-procedural evolution, illustrating its dual diagnostic and interventional utility. Additional support for expanding brain POCUS applications comes from two illustrative case reports by Oulego-Erroz (2020) and Lange (2021) [17,18]. Both studies demonstrated that incorporating mastoid, posterolateral, and posterior fossa views can reveal lesions that are not accessible through standard anterior coronal and sagittal planes. This extended scanning strategy may therefore play a decisive role in detecting posterior fossa and midline pathologies in time-critical scenarios, broadening the operational value of brain POCUS beyond its conventional applications.

#### Synthesis of Outcomes Relevant to an Emergency Brain POCUS Protocol

Across the nine included studies, several reproducible elements emerged that can inform the development of a structured brain POCUS protocol for neonatal and pediatric emergency and critical care. These components provide the operational backbone of the proposed KORE Brain POCUS Protocol:

1. Core scanning windows: Routine anterior fontanelle coronal and sagittal sweeps should be systematically performed, with deliberate inclusion of mastoid and posterolateral views when posterior fossa or midline pathology is suspected [10,12,17,18].
2. Structured measurements: In preterm infants at risk of PHVD/IVH, serial assessment of VI, AHW, and FOHR/FTHR ratios against gestational-age–specific reference values allows early recognition of progressive disease and timely neurosurgical discussion [12].
3. Hemodynamic adjuncts: When cerebral perfusion impairment or raised ICP is suspected, transcranial Doppler interrogation of MCA, ACA, ICA, or BA with age-appropriate interpretation of PI and RI provides valuable physiological insight [10,11].
4. Procedural applications: POCUS can be used to guide bedside hematoma aspiration in unstable neonates, supporting both patient selection and procedural monitoring [13].
5. Operational feasibility in the emergency department: Pediatric Emergency Medicine (PEM)-led cranial POCUS has shown the capacity to detect ICH rapidly in young infants, potentially reducing transfers and CT exposure, provided operators have targeted training and escalation pathways [14].
6. Integration into sepsis pathways: Cranial ultrasound can be embedded into multi-organ POCUS workflows to identify neurological complications during late-onset sepsis evaluations [15].

#### Heterogeneity and Evidence Gaps

**Figure 2.**
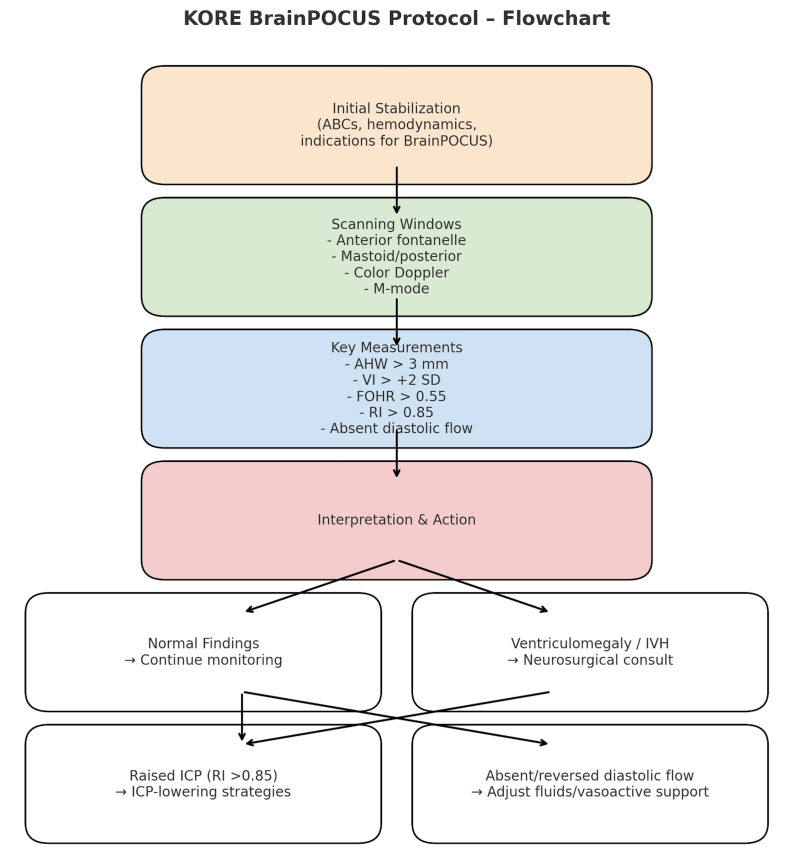
KORE Brain POCUS Protocol. Flowchart summarizing the stepwise approach to cranial point of care ultrasound in neonates and infants. The protocol integrates initial stabilization, scanning windows, key ventricular and Doppler measurements, and interpretation with corresponding clinical actions. AHW = anterior horn width; FOHR = frontal–occipital horn ratio; ICP = intracranial pressure; IVH = intraventricular hemorrhage; RI = resistive index; VI = ventricular index.

Marked methodological heterogeneity, including mixed study designs, small prospective cohorts, and several single-patient case reports, precluded any formal meta-analysis. Nevertheless, the convergence of findings across diverse designs supports the overall feasibility and clinical utility of brain POCUS in neonatal and pediatric urgent care settings. The most developed quantitative evidence currently pertains to PHVD surveillance, for which standardized measurement thresholds have been established [12]. In contrast, prospective diagnostic-accuracy data from the pediatric emergency department remain limited to small pilot studies [14]. Multiple narrative reviews consistently emphasize the need for structured training, competency assessment, and the development of outcome-linked protocols to ensure reliable and reproducible implementation [10,11]. Building on these convergent findings, we synthesized the evidence into the KORE Brain POCUS Protocol, a structured bedside algorithm summarizing the recommended scanning sequence, key measurements, and associated clinical actions (Figure 2, Table 1). The KORE Brain POCUS Protocol is designed to provide a structured, stepwise approach to the bedside evaluation of neonates and young infants presenting with acute neurological concerns. It integrates standardized scanning windows, key measurements, and clinical decision points to support rapid diagnosis and management.

**Table 1.** Proposed KORE Brain POCUS Protocol for Neonates and Infants. ABCs, airway, breathing, circulation; ACA, anterior cerebral artery; AHW, anterior horn width; BA, basilar artery; FOHR, frontal–occipital horn ratio; ICA, internal carotial artery; ICP, intracranial pressure; IVH, intraventricular hemorrhage; MCA, middle cerebral artery; RI, resistive index; VI, ventricular index. Note on measurements: All measurement thresholds included in the KORE Brain POCUS Protocol are derived from published literature and reflect values most frequently cited in neonatal and pediatric neurosonography studies [10–12,15]. These cut-offs are not newly validated by the present review but are synthesized from available evidence to support standardized bedside interpretation and early clinical decision-making.

Steps	Content
1. Initial Stabilization	Assess ABCs and hemodynamic status Indications: altered mental status, bulging fontanelle, seizures, head trauma, suspected IVH/hydrocephalus
2. Scanning Windows & Modalities	Anterior fontanelle (coronal, sagittal) → ventricles, parenchyma Mastoid/posterior fontanelle → posterior fossa, cerebellum Color Doppler → ACA, MCA, ICA, BA M-mode → midline shift
3. Key Measurements (suggested cut-offs)	AHW > 3 mm → risk of IVH/ventriculomegaly (preterm) VI > +2 SD for age → ventricular dilatation FOHR > 0.55 → hydrocephalus RI > 0.85 → raised ICP
4. Interpretation & Clinical Action	Normal findings → continue monitoring Ventriculomegaly / IVH → notify neonatology / neurosurgery High ICP markers (RI↑, AHW↑, VI↑) → initiate ICP-lowering strategies, escalate care Hemodynamic compromise (RI >0.85, absent diastolic flow) → adjust fluids/vasoactive support

Step 1 – Initial Stabilization. The first priority is to assess airway, breathing, and circulation (ABCs) and ensure hemodynamic stability. Typical indications for initiating brain POCUS include altered mental status, bulging fontanelle, seizures, head trauma, and suspected IVH or hydrocephalus. Early stabilization ensures safe and meaningful imaging.

Step 2 – Scanning Windows and Modalities. Imaging begins with the anterior fontanelle, performing coronal and sagittal sweeps to evaluate ventricular size, parenchymal structure, and midline integrity. Mastoid and posterior fontanelle views are added when posterior fossa or cerebellar pathology is suspected. Colour Doppler interrogation of the ACA, MCA, ICA, and BA provides complementary hemodynamic information, while M-mode can be used to assess midline shift dynamically.

Step 3 – Key Measurements. To support structured interpretation, several measurements can be obtained at the bedside:

AHW > 3 mm suggests ventriculomegaly or IVH in preterm infants.

VI > +2 SD for gestational age indicates progressive ventricular dilatation.

FOHR > 0.55 is consistent with hydrocephalus.

RI > 0.85 raises concern for elevated ICP.

Absent or reversed diastolic flow reflects critical cerebral perfusion compromise.

Step 4 – Interpretation and Clinical Action. Normal findings warrant continued observation and, when clinically appropriate, serial monitoring. Ventriculomegaly, IVH, or other structural abnormalities should prompt early communication with neonatology and neurosurgery teams and timely escalation to confirmatory neuroimaging (CT or MRI) when indicated. Doppler abnormalities suggestive of impaired cerebral perfusion should be interpreted cautiously in the clinical context and may support the need for urgent specialist consultation and further diagnostic evaluation. These decision steps are summarized as a structured bedside triage framework (Table 2).

**Table 2.** Rapid Cut-off Values for KORE Brain POCUS (Pocket Reference). AHW, anterior horn width; FOHR, frontal–occipital horn ratio; PI, pulsatility index; PHVD, post-hemorrhagic ventricular dilation; RI, resistive index; TOD, thalamo-occipital distance; VI, ventricular index. Note on Doppler interpretation: In term and preterm neonates, commonly reported RI values are around ∼0.70 in the first 24 hours and may decline with maturation; interpretation should prioritize serial trends and account for systemic factors (PaCO_2_, mean arterial pressure, hemoglobin, temperature). Adult PI/RI thresholds should not be extrapolated to neonates; use age-aware references and clinical context [10,12,19].

Index	Alert Threshold	Probable Action Threshold	Notes
AHW	≥ 6 mm	≥ 10 mm	High reproducibility; strong predictor of PHVD progression.
VI (Levene)	≥ p97	≥ p97 + 4 mm	Age-specific; use reference curves.
FOHR	≥ 0.50 (referral)	≥ 0.55 (frequent intervention)	Correlates with ventricular volumes and outcome; ≥0.60–0.65 = high risk.
TOD	—	> 25 mm	May precede anterior horn enlargement.
Doppler	End-diastolic velocity ↓ / absent / reversed; RI/PI ↑	Arrest patterns (reverberating, spikes)	Use intra-patient trend; integrate with clinical/ near-infrared spectroscopy.

## Discussion

This structured evidence synthesis demonstrates that brain POCUS in neonates and children is technically feasible, repeatable at the bedside, and clinically informative across three key domains relevant to acute care: (1) surveillance of ventricular size and hemorrhage (screening and follow-up of IVH/ventriculomegaly), (2) assessment of cerebral hemodynamics (Doppler-based trends in flow velocity and impedance, particularly in unstable physiology), and (3) detection of anatomic “red flags” (e.g., hydrocephalus, extra-axial collections, midline shifts) that can support triage to advanced imaging or prompt neurosurgical consultation. Although the nine included studies were heterogeneous in design and setting (NICU, PICU, intraoperative, and emergency department/acute care), they converge on a consistent signal: Brain POCUS can shorten the time from clinical deterioration to actionable information when the open fontanelle provides an optimal acoustic window, and Doppler is applied with disciplined technique. In neonates and young infants, the anterior fontanelle allows high-quality 2D and Doppler insonation of the ACA, MCA, ICA, and BA with standard portable ultrasound equipment. This aligns with existing neonatal standards and intraoperative literature where transfontanelle ultrasonography has been used to guide decision-making in real time; for example, during cardiac surgery or when cerebral oximetry drops [20]. In older children with closed fontanelles, the evidence is more limited and focuses on transtemporal Doppler windows, consistent with broader transcranial Doppler-based pediatric literature [19]. Importantly, diagnostic feasibility and accuracy decline in late infancy as fontanelles progressively close and acoustic windows become less reliable. Therefore, the KORE Brain POCUS Protocol is expected to perform best in neonates and early infancy, while in older infants, its role should remain limited to selected cases and adjunct assessment. Routine NICU cranial ultrasound protocols establish reliable linear indices such as AHW, VI, and complementary ratios (FOHR/FTHR) that predict disease progression and the need for temporizing cerebral spinal fluid diversion. Importantly, the direction of change over time is more informative than a single absolute value, underscoring the role of serial assessments. This “measure-and-trend” approach is central to the proposed KORE Brain POCUS Protocol and aligns with contemporary neonatal standards [12]. Bedside Doppler adds physiological information to anatomic imaging. Peak systolic velocity, end-diastolic velocity, and derived PI and RI reflect the dynamic balance between cardiac output, PaCO_2_, perfusion pressure, and intracranial/venous pressures. Pediatric intraoperative series show that transfontanelle ultrasonography can detect clinically relevant fluctuations in cerebral brain fluid velocity during anesthesia and cardiopulmonary bypass, and guide management in combination with near-infrared spectroscopy [20]. Compared to transtemporal transcranial Doppler, commonly used in adults, the transfontanelle approach offers an optimized insonation angle and improved waveform quality, which is directly translatable to emergency monitoring. Brain POCUS reliably identifies hydrocephalus, intraventricular clots, and extra-axial collections in infants. Doppler detection of high-intensity transient signals has been described intra-operatively, but their clinical significance in pediatrics remains uncertain and should not currently guide independent interventions [19]. In most emergency settings, CT remains the preferred first-line modality when intracranial pathology is strongly suspected. The feasibility of performing comprehensive Doppler-based brain POCUS prior to CT is likely limited to selected stable infants with open fontanelles and immediate operator availability, and it should not delay definitive neuroimaging when urgently indicated. Brain POCUS should therefore be positioned as a rapid bedside triage tool that may accelerate escalation to advanced neuroimaging, neurosurgical consultation, or transfer to higher-level centers, rather than as a replacement for CT or MRI. POCUS has transformed emergency and critical care by introducing structured, high-yield protocols (eFAST, RUSH, BLUE) that sharpen pre-test probabilities and accelerate definitive care. Despite robust neonatal cranial ultrasound standards and growing perioperative evidence, a structured, triage-oriented brain POCUS pathway for emergency pediatrics remains lacking. This synthesis provides sufficient face validity and operational feasibility to justify codification of a protocol, with the important caveat that diagnostic accuracy and patient-centred outcomes still require prospective validation. Building on the evidence above, the KORE Brain POCUS Protocol addresses four time-critical questions that influence immediate management:

*Is ventricular pressure/volume likely elevated?* Core views include coronal, parasagittal, and posterior horn sweeps; key measures are AHW, VI, and optional FOHR/FTHR. An early neurosurgical consult should be considered when morphology and clinical signs diverge. Serial measurements are more informative than static cut-offs.

*Is cerebral perfusion plausibly threatened?* Doppler interrogation of ACA/MCA/ICA (peak systolic velocity, end-diastolic velocity, PI, RI) should be interpreted in relation to systemic changes (mean arterial pressure, PaCO_2_, ventilation, positioning, sedation). Actions include titrating cardiorespiratory support and repeating scans after interventions.

*Is there an urgent structural lesion?* Standard 2D scanning identifies IVH, extra-axial collections, and midline shift. Concerning findings should expedite neuroimaging or escalation of care.

Three pragmatic anchors emerged from the review and can inform the safe and effective implementation of brain POCUS in neonatal and pediatric acute care. First, standardizing image acquisition is essential to preserve measurement reliability over time. This includes maintaining an angle correction of less than 20 degrees, ensuring consistent insonation depth, and acquiring images on the same anatomical planes during serial examinations. These measures help protect the integrity of trend-based interpretations, which are central to the proposed protocol. Second, it is important to teach measurement discipline. Examiners should prioritize obtaining AHW and VI as the primary quantitative parameters, and reserve ratios such as FOHR/FTHR for situations in which ventricular geometry is distorted or more complex. Precise documentation of the insonation plane is equally critical to ensure reproducibility across different operators and time points. Finally, brain POCUS findings should always be interpreted in the clinical context. Morphological and Doppler data should be triangulated with hemodynamic parameters, blood gas analyses, neurological examination, and, where available, near-infrared spectroscopy. Together, these principles form the foundation for consistent and safe clinical application.

As with other successful POCUS protocols, structured training, inter-operator reliability assessment, and multicenter validation will be essential to ensure reproducibility and scalability in real-world practice. The findings of this review suggest that the field is ready to move beyond feasibility and towards structured validation of brain POCUS in neonatal and pediatric acute care. Several key research directions emerge as particularly relevant. There is a clear need for prospective, multicenter diagnostic accuracy studies comparing the KORE Brain POCUS Protocol with reference standards such as MRI, CT, or comprehensive NICU cranial ultrasound. These studies should address predefined emergency questions, including the detection of clinically significant ventriculomegaly, large extra-axial collections, and major IVH. Physiology-to-outcome trials are required to determine whether Doppler-guided titration of ventilation and vasoactive therapy can meaningfully reduce secondary brain injury. Relevant endpoints may include the frequency of severe desaturation events, the need for rescue CT imaging, and time to neurosurgical decision-making. Furthermore, establishing measurement reliability is essential. Inter- and intra-rater agreement for key indices such as AHW, VI, and FOHR should be evaluated in real-world emergency and critical care settings to define competency thresholds for credentialing. Research on health service outcomes could clarify the system-level impact of brain POCUS, including its effect on time to diagnosis, radiation exposure, patient transfers, and cost-effectiveness in both high-resource and resource-limited environments. Finally, protocol-refinement studies should delineate when to incorporate adjunct modalities—such as optic nerve sheath diameter assessment in older infants or transtemporal transcranial Doppler when feasible—and when to escalate directly to advanced imaging.

Within its current evidence boundaries, brain POCUS structured by the KORE Brain POCUS Protocol offers a rapid and physiologically anchored framework for the first critical hour of neonatal and early pediatric neurological emergencies. It is not intended to replace comprehensive imaging or neuromonitoring. Instead, its role is to front-load high-value diagnostic information, support triage decisions, guide cardio-respiratory management, and determine who requires advanced imaging, and with what degree of urgency. The convergence between the nine included studies and existing neonatal cranial ultrasound standards supports the feasibility of implementation, provided that operators receive appropriate training and that quality assurance systems are in place. A coordinated prospective validation will be essential to confirm its diagnostic accuracy, clinical impact, and scalability.

## Limitations

This review has several limitations that must be acknowledged. First, the number of eligible studies was relatively small, reflecting the nascent nature of this field. Most of the included reports were narrative reviews, case series, or observational studies, with a paucity of randomized or prospective controlled data. Second, there was marked heterogeneity across studies in terms of populations, clinical settings, ultrasound equipment, operators' expertise, and outcome measures, which limited the ability to conduct a formal meta-analysis. Third, publication bias cannot be excluded, as studies with negative or inconclusive findings may be underrepresented. Finally, although the proposed KORE Brain POCUS Protocol is derived from converging evidence, it remains conceptual and requires rigorous validation before it can be considered a standardized clinical tool.

## Conclusion

Cranial POCUS has emerged as a promising modality for the rapid, bedside assessment of critically ill neonates and pediatric patients. The available evidence, although limited, consistently highlights its value in diagnosing IVH, hydrocephalus, elevated ICP, and in guiding clinical decision-making in acute and intensive care settings. To date, however, no structured protocol equivalent to established frameworks such as BLUE or RUSH exists for pediatric neurocritical care. Based on this systematic synthesis, we propose the KORE Brain POCUS Protocol as a conceptual framework to standardize and integrate cranial POCUS into emergency and critical care pathways. Future research should prioritize multicenter, prospective trials to validate the diagnostic accuracy, reproducibility, and clinical impact of brain POCUS in neonatal and pediatric emergencies. Standardization of image acquisition, interpretation criteria, and operator training is urgently needed. Comparative studies evaluating brain POCUS against conventional neuroimaging modalities, as well as its integration with multimodal neuromonitoring, will be essential to establish its role in routine critical care practice. Ultimately, implementing a validated Brain POCUS protocol could improve diagnostic timeliness, optimize resource utilization, and enhance outcomes in vulnerable pediatric populations.
